# 2,4-Diiodo-6-[(propyl­imino)­meth­yl]phenol

**DOI:** 10.1107/S1600536812005727

**Published:** 2012-02-24

**Authors:** Peng-Gang Liu, Xiao-Ning Wang, Yong-An Yang, Hai-Liang Zhu

**Affiliations:** a State Key Laboratory of Pharmaceutical Biotechnology, Nanjing University, Nanjing 210093, People’s Republic of China

## Abstract

The title compound, C_10_H_11_I_2_NO, was prepared by the reaction of 3,5-diiodo­salicyl­aldehyde with propyl­amine in ethanol. The mol­ecule adopts an *E* conformation with respect to the C=N bond and the aromatic ring. The aromatic ring and the imino unit are close to being coplanar, with a dihedral angle of 2.6 (3)° between their planes. This planarity is assisted by the formation of an intra­molecular O—H⋯O hydrogen bond.

## Related literature

For the biological activity of Schiff base compounds, see: Chohan *et al.* (2012 ▶); Yan *et al.* (2011 ▶); Zhang *et al.* (2011 ▶). For their use as ligands in coordination chemistry, see: You *et al.* (2008 ▶); Xu *et al.* (2009 ▶); Chen *et al.* (2010 ▶); Cui *et al.* (2011 ▶). For standard bond distances, see: Allen *et al.* (1987 ▶).
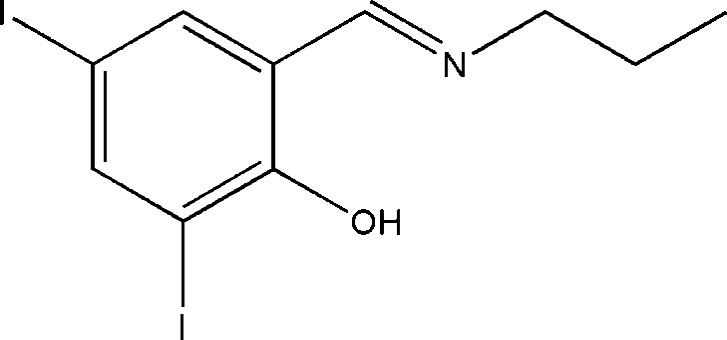


## Experimental

### 

#### Crystal data

C_10_H_11_I_2_NO
*M* *_r_* = 415.00
Orthorhombic, 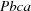

*a* = 10.7019 (14) Å
*b* = 7.1483 (9) Å
*c* = 32.404 (4) Å
*V* = 2478.9 (5) Å^3^
*Z* = 8
Mo *K*α radiation
μ = 5.05 mm^−1^
*T* = 298 K
0.21 × 0.20 × 0.20 mm

#### Data collection

Bruker SMART CCD area-detector diffractometer
Absorption correction: multi-scan (*SADABS*; Sheldrick, 1996 ▶) *T*_min_ = 0.417, *T*_max_ = 0.432
18976 measured reflections
2704 independent reflections
2224 reflections with *I* > 2σ(*I*)
*R*_int_ = 0.030

#### Refinement

*R*[*F* ^2^ > 2σ(*F* ^2^)] = 0.035
*wR*(*F* ^2^) = 0.104
*S* = 1.23
2704 reflections
131 parameters
1 restraint
H atoms treated by a mixture of independent and constrained refinement
Δρ_max_ = 0.96 e Å^−3^
Δρ_min_ = −0.89 e Å^−3^

### 

Data collection: *SMART* (Bruker, 1998 ▶); cell refinement: *SAINT* (Bruker, 1998 ▶); data reduction: *SAINT*; program(s) used to solve structure: *SHELXS97* (Sheldrick, 2008 ▶); program(s) used to refine structure: *SHELXL97* (Sheldrick, 2008 ▶); molecular graphics: *SHELXTL* (Sheldrick, 2008 ▶); software used to prepare material for publication: *SHELXTL*.

## Supplementary Material

Crystal structure: contains datablock(s) global, I. DOI: 10.1107/S1600536812005727/sj5194sup1.cif

Structure factors: contains datablock(s) I. DOI: 10.1107/S1600536812005727/sj5194Isup2.hkl

Supplementary material file. DOI: 10.1107/S1600536812005727/sj5194Isup3.cml

Additional supplementary materials:  crystallographic information; 3D view; checkCIF report

## Figures and Tables

**Table 1:** Hydrogen-bond geometry (Å, °)

*D*—H⋯*A*	*D*—H	H⋯*A*	*D*⋯*A*	*D*—H⋯*A*
O1—H1⋯N1	0.90 (1)	1.82 (5)	2.567 (6)	138 (7)

**Fig. 1.**
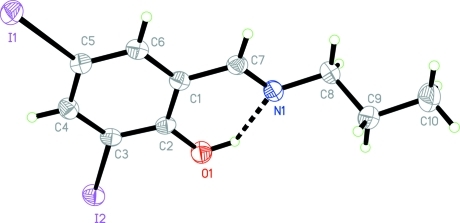
The molecular structure of the title compound, showing the atom labelling scheme. Displacement ellipsoids are drawn at the 30% probability level. An intramolecular hydrogen bond is indicated by a dashed line.

## supplementary crystallographic information

### Comment

Schiff bases have been extensively studied because of their biological activity (Chohan *et al.*, 2012; Yan *et al.*, 2011; Zhang *et al.*, 2011). In addition, Schiff bases have been shown to be versatile ligands for the preparation of coordination complexes (You *et al.*, 2008; Xu *et al.*, 2009; Chen *et al.*, 2010; Cui *et al.*, 2011). In the present paper, the structure of the new title Schiff base compound is reported.

The molecule of the compound exists in a *trans* of *E* configuration with respect to the methylidene unit. The torsion angles C1—C7—N1—C8, C7—N1—C8—C9, and N1—C8—C9—C10 are 0.9 (2), 60.5 (2), and 4.6 (2)°, respectively. Bond distances are within normal values (Allen *et al.*, 1987). An intramolecular O1—H1···N1 hydrogen bond stabilises the molecular structure.

### Experimental

3,5-Diiodosalicylaldehyde (0.37 g, 1 mmol) and propylamine (0.06 g, 1 mmol) were mixed in ethanol (20 ml). The mixture was stirred at room temperature for 30 min to give a yellow solution. Yellow block-shaped single crystals were obtained by slow evaporation of this solution in air.

### Refinement

H1 was located from a difference Fourier map and refined isotropically, with the O—H distance restrained to 0.90 (1) Å. The remaining H-atoms were positioned geometrically and refined using a riding model, with C—H = 0.93–0.97 Å, and with *U*_iso_(H) set to 1.2*U*_eq_(C) and 1.5*U*_eq_(C10).

### Crystal data

**Table:**

C_10_H_11_I_2_NO	*F*(000) = 1536
*M**_r_* = 415.00	*D*_x_ = 2.224 Mg m^−^^3^
Orthorhombic, *P**b**c**a*	Mo *K*α radiation, λ = 0.71073 Å
Hall symbol: -P 2ac 2ab	Cell parameters from 1027 reflections
*a* = 10.7019 (14) Å	θ = 2.3–24.5°
*b* = 7.1483 (9) Å	µ = 5.05 mm^−^^1^
*c* = 32.404 (4) Å	*T* = 298 K
*V* = 2478.9 (5) Å^3^	Block, yellow
*Z* = 8	0.21 × 0.20 × 0.20 mm

### Data collection

**Table:**

Bruker SMART CCD area-detector diffractometer	2704 independent reflections
Radiation source: fine-focus sealed tube	2224 reflections with *I* > 2σ(*I*)
Graphite monochromator	*R*_int_ = 0.030
ω scans	θ_max_ = 27.0°, θ_min_ = 2.3°
Absorption correction: multi-scan (*SADABS*; Sheldrick, 1996)	*h* = −13→13
*T*_min_ = 0.417, *T*_max_ = 0.432	*k* = −9→8
18976 measured reflections	*l* = −41→41

### Refinement

**Table:**

Refinement on *F*^2^	Primary atom site location: structure-invariant direct methods
Least-squares matrix: full	Secondary atom site location: difference Fourier map
*R*[*F*^2^ > 2σ(*F*^2^)] = 0.035	Hydrogen site location: inferred from neighbouring sites
*wR*(*F*^2^) = 0.104	H atoms treated by a mixture of independent and constrained refinement
*S* = 1.23	*w* = 1/[σ^2^(*F*_o_^2^) + (0.038*P*)^2^ + 7.4143*P*] where *P* = (*F*_o_^2^ + 2*F*_c_^2^)/3
2704 reflections	(Δ/σ)_max_ < 0.001
131 parameters	Δρ_max_ = 0.96 e Å^−^^3^
1 restraint	Δρ_min_ = −0.89 e Å^−^^3^

### Special details

**Table:**

Geometry. All e.s.d.'s (except the e.s.d. in the dihedral angle between two l.s. planes) are estimated using the full covariance matrix. The cell e.s.d.'s are taken into account individually in the estimation of e.s.d.'s in distances, angles and torsion angles; correlations between e.s.d.'s in cell parameters are only used when they are defined by crystal symmetry. An approximate (isotropic) treatment of cell e.s.d.'s is used for estimating e.s.d.'s involving l.s. planes.
Refinement. Refinement of *F*^2^ against ALL reflections. The weighted *R*-factor *wR* and goodness of fit *S* are based on *F*^2^, conventional *R*-factors *R* are based on *F*, with *F* set to zero for negative *F*^2^. The threshold expression of *F*^2^ > σ(*F*^2^) is used only for calculating *R*-factors(gt) *etc*. and is not relevant to the choice of reflections for refinement. *R*-factors based on *F*^2^ are statistically about twice as large as those based on *F*, and *R*- factors based on ALL data will be even larger.

### Fractional atomic coordinates and isotropic or equivalent isotropic displacement parameters (Å^2^)

**Table:**

	*x*	*y*	*z*	*U*_iso_*/*U*_eq_	
I1	0.14060 (4)	0.18844 (7)	0.522554 (12)	0.05384 (15)	
I2	−0.14147 (3)	0.44774 (7)	0.371421 (13)	0.05752 (16)	
N1	0.3755 (4)	0.4405 (7)	0.34042 (16)	0.0449 (11)	
O1	0.1359 (4)	0.4554 (7)	0.33939 (13)	0.0520 (10)	
C1	0.2521 (5)	0.3571 (7)	0.39908 (16)	0.0378 (11)	
C2	0.1382 (5)	0.3981 (8)	0.37824 (17)	0.0393 (11)	
C3	0.0266 (5)	0.3772 (7)	0.40084 (16)	0.0396 (11)	
C4	0.0250 (5)	0.3193 (8)	0.44167 (17)	0.0444 (12)	
H4	−0.0501	0.3087	0.4559	0.053*	
C5	0.1389 (5)	0.2770 (7)	0.46109 (17)	0.0397 (11)	
C6	0.2501 (5)	0.2976 (7)	0.44023 (16)	0.0415 (11)	
H6	0.3250	0.2716	0.4537	0.050*	
C7	0.3699 (5)	0.3819 (8)	0.37775 (18)	0.0431 (12)	
H7	0.4438	0.3544	0.3916	0.052*	
C8	0.4981 (6)	0.4653 (9)	0.32071 (19)	0.0520 (14)	
H8A	0.5636	0.4343	0.3402	0.062*	
H8B	0.5085	0.5952	0.3127	0.062*	
C9	0.5099 (6)	0.3412 (10)	0.28282 (19)	0.0572 (16)	
H9A	0.4486	0.3791	0.2624	0.069*	
H9B	0.4926	0.2126	0.2904	0.069*	
C10	0.6403 (7)	0.3541 (14)	0.2642 (2)	0.077 (2)	
H10A	0.6567	0.4809	0.2561	0.115*	
H10B	0.6453	0.2740	0.2405	0.115*	
H10C	0.7009	0.3154	0.2843	0.115*	
H1	0.211 (3)	0.471 (11)	0.327 (2)	0.080*	

### Atomic displacement parameters (Å^2^)

**Table:**

	*U*^11^	*U*^22^	*U*^33^	*U*^12^	*U*^13^	*U*^23^
I1	0.0536 (2)	0.0651 (3)	0.0428 (2)	−0.0002 (2)	−0.00119 (15)	0.00945 (18)
I2	0.0393 (2)	0.0751 (3)	0.0582 (3)	0.00399 (19)	−0.01085 (16)	0.0073 (2)
N1	0.039 (2)	0.045 (3)	0.050 (3)	−0.002 (2)	0.0032 (19)	−0.001 (2)
O1	0.048 (2)	0.060 (3)	0.048 (2)	0.004 (2)	−0.0015 (17)	0.008 (2)
C1	0.037 (2)	0.032 (3)	0.045 (3)	0.003 (2)	−0.002 (2)	−0.005 (2)
C2	0.044 (3)	0.035 (3)	0.040 (3)	0.001 (2)	−0.004 (2)	−0.006 (2)
C3	0.039 (3)	0.035 (3)	0.045 (3)	0.004 (2)	−0.008 (2)	−0.002 (2)
C4	0.040 (3)	0.042 (3)	0.052 (3)	0.002 (2)	0.002 (2)	−0.002 (2)
C5	0.045 (3)	0.033 (3)	0.042 (3)	0.002 (2)	−0.003 (2)	0.001 (2)
C6	0.040 (3)	0.036 (3)	0.048 (3)	0.004 (2)	−0.005 (2)	−0.003 (2)
C7	0.041 (3)	0.043 (3)	0.045 (3)	0.002 (2)	−0.002 (2)	−0.009 (2)
C8	0.041 (3)	0.059 (4)	0.057 (3)	−0.006 (3)	0.005 (2)	0.000 (3)
C9	0.057 (4)	0.067 (4)	0.048 (3)	−0.006 (3)	0.005 (3)	−0.001 (3)
C10	0.070 (5)	0.099 (6)	0.061 (4)	−0.003 (4)	0.017 (3)	0.005 (4)

### Geometric parameters (Å, º)

**Table:**

I1—C5	2.090 (5)	C5—C6	1.377 (7)
I2—C3	2.097 (5)	C6—H6	0.9300
N1—C7	1.282 (8)	C7—H7	0.9300
N1—C8	1.471 (7)	C8—C9	1.520 (9)
O1—C2	1.324 (7)	C8—H8A	0.9700
O1—H1	0.900 (10)	C8—H8B	0.9700
C1—C6	1.400 (7)	C9—C10	1.523 (9)
C1—C2	1.424 (7)	C9—H9A	0.9700
C1—C7	1.449 (7)	C9—H9B	0.9700
C2—C3	1.409 (7)	C10—H10A	0.9600
C3—C4	1.386 (8)	C10—H10B	0.9600
C4—C5	1.405 (7)	C10—H10C	0.9600
C4—H4	0.9300		
			
C7—N1—C8	119.4 (5)	N1—C7—H7	119.0
C2—O1—H1	116 (5)	C1—C7—H7	119.0
C6—C1—C2	120.1 (5)	N1—C8—C9	110.7 (5)
C6—C1—C7	120.3 (5)	N1—C8—H8A	109.5
C2—C1—C7	119.6 (5)	C9—C8—H8A	109.5
O1—C2—C3	120.7 (5)	N1—C8—H8B	109.5
O1—C2—C1	122.1 (5)	C9—C8—H8B	109.5
C3—C2—C1	117.2 (5)	H8A—C8—H8B	108.1
C4—C3—C2	122.6 (5)	C8—C9—C10	111.1 (6)
C4—C3—I2	119.7 (4)	C8—C9—H9A	109.4
C2—C3—I2	117.7 (4)	C10—C9—H9A	109.4
C3—C4—C5	118.7 (5)	C8—C9—H9B	109.4
C3—C4—H4	120.6	C10—C9—H9B	109.4
C5—C4—H4	120.6	H9A—C9—H9B	108.0
C6—C5—C4	120.5 (5)	C9—C10—H10A	109.5
C6—C5—I1	119.5 (4)	C9—C10—H10B	109.5
C4—C5—I1	120.0 (4)	H10A—C10—H10B	109.5
C5—C6—C1	120.9 (5)	C9—C10—H10C	109.5
C5—C6—H6	119.6	H10A—C10—H10C	109.5
C1—C6—H6	119.6	H10B—C10—H10C	109.5
N1—C7—C1	122.1 (5)		

### Hydrogen-bond geometry (Å, º)

**Table:**

*D*—H···*A*	*D*—H	H···*A*	*D*···*A*	*D*—H···*A*
O1—H1···N1	0.90 (1)	1.82 (5)	2.567 (6)	138 (7)
